# Effect of Heparin on Prevention of Flap Loss in Microsurgical Free Flap Transfer: A Meta-Analysis

**DOI:** 10.1371/journal.pone.0095111

**Published:** 2014-04-21

**Authors:** Xuan-liang Pan, Guo-xian Chen, Hua-wei Shao, Chun-mao Han, Li-ping Zhang, Li-zhu Zhi

**Affiliations:** Department of Burns, Second Affiliated Hospital of Zhejiang University School of Medicine, Hangzhou, Zhejiang, China; University of Rome, Italy

## Abstract

The effectiveness of heparin for thromboprophylaxis during microvascular free flap transfer is uncertain. The purpose of this meta-analysis was to determine the effect of heparin on the prevention of flap loss in microsurgical free flap transfer.A search of PubMed, Cochrane databases, and Google Scholar using combinations of the search terms heparin, free flap, flap loss, free tissue transfer was conducted on March 15, 2013. Inclusion criteria were: 1) Prospective randomized trials. 2) Retrospective, non-randomized studies. 3) Patients received free tissue transfer. Flap loss rate was used to evaluate treatment efficacy. Odds ratios (ORs) with 95% confidence intervals (CI) were calculated and compared between therapies. Four studies meet the criteria for analysis and were included. Two studiescompared aspirin and heparin, and the ORs of the 2 studies were 1.688 and 2.087. The combined OR of 2.003 (95% CI 0.976–4.109, *p* = 0.058) did not indicate any significant difference between heparin and aspirin therapies. Two studiescompared high and low doses of dalteparin/heparin therapies, and the ORs of the 2 studies were 4.691 and 11.00. The combined OR of 7.810 (95% CI 1.859–32.808, *p* = 0.005) revealed a significant difference indicating that high dose dalteparin or heparin therapy is associated with a greater flap loss rate than low dose therapy. Heparin and aspirin prophylaxis are associated with similar flap loss rates after free flap transfer, and high dose dalteparin or heparin therapy is associated with a greater flap loss rate than low dose therapy.

## Introduction

Microvascular free tissue transfer has revolutionized reconstructive surgery, and a multitude of surgical flaps available to meet the needs of the recipient site [1]. Flap success rates range from 90 to 99% [2], and surgeon experience has been reported to be one of the most important factors associated with flap survival [3]. Despite improvements in surgical technique and methods, when small vessels are anastomosed, there is a risk of thrombotic occlusion, which is the leading cause of flap failure [4],[5].

To reduce the possibility of thrombotic occlusion after free flap transfer, and thus the possibility of flap failure, anticoagulants are frequently administered [4],[6],[7]. Most regimens for the prophylaxis against thrombosis and flap failure use aspirin, low-molecular-weight heparin (LMWH), or colloids such as dextran [4],[5]. While many studies have examined various methods of preventing thrombosis and flap failure, clear evidence is lacking on the efficacy, dosage, and timing of anticoagulant agents available for the prevention and treatment of thrombosis in microvascular surgery and no consensus has been achieved [8]. Furthermore, all anticoagulants carry the risk of bleeding as well as other sometimes serious side effects.

LMWH is routinely used for thromboprophylaxis in various surgical procedures [9]–[11]. It is also one of the most commonly used agents for prophylaxis against thrombosis in free flap transfer [7],[12],[13]. Various dosages and timing of administration have been described for the use of LMWH to prevent flap loss after free flap transfer; however, evidence is lacking as to the effectiveness of any particular regimen, or even the use of LMWH.

Thus, the objective of this meta-analysis was to evaluate the effect of heparin on flap loss after free tissue transfer.

## Methods

### Literature Search Strategy

A search was conducted of PubMed, Cochrane databases, and Google Scholar using combinations of the search terms: heparin, free flap, flap loss, free tissue transfer. The search was conducted March 15, 2013. Each publication was carefully examined, including the names of all authors, to avoid duplication of data.

### Selection Criteria

Studies were selected for inclusion in this analysis based on the following criteria. 1) 1) Prospective randomized trials. 2) Retrospective, non-randomized studies. 3) Patients who received free tissue transfer. 4) Evaluated heparin for the prevention of flap ischemia-reperfusion injury. Exclusion criteria for this analysis were as follows. 1) Studies that evaluate the heparin used to salvage compromised flaps. 2) No information of flap loss was provided. 3) Non-human studies. 4) Case reports, case series without comparison groups, letters, and editorials.

### Data Extraction

Two independent reviewers extracted the data from eligible studies. A third reviewer was consulted for resolution of any disagreement. Data extracted included type of study, method of heparin administration and dosage, number of patients and demographic and clinical data, flap loss, the occurrence of hematoma formation and hemorrhage, and overall complication rate. The primary outcome measure was flap loss rate.

### Data Analysis

The primary outcome, flap loss rate, was used to evaluate treatment efficacy. Odds ratios (ORs) with 95% confidence intervals (CI) were calculated for binary outcome data, and were compared between therapies. A χ^2^-based test of homogeneity was performed, and the inconsistency index (I^2^) statistic was determined. If I^2^ was >50% or >75%, the trials were considered to be heterogeneous or highly heterogeneous, respectively. If I^2^ was <25%, the studies were considered to be homogeneous. If the I^2^ statistic (>50%) indicated heterogeneity existed between studies, a random-effects model was calculated. Otherwise, fixed-effects models were calculated. Pooled summary statistics for the ORs of the individual studies were reported. A *p*-value of less than 0.05 was chosen to indicate statistical significance. Sensitivity analysis based on the leave-one-out approach was not performed because only 2 studies were considered for each analysis. Similarly, publication bias analysis was not performed because the limitation of a small number of studies did not allow detection of an asymmetric funnel [14]. All analyses were performed using Comprehensive Meta-Analysis statistical software, version 2.0 (Biostat, Englewood, NJ).

## Results

### Literature Search

**Figure 1:**
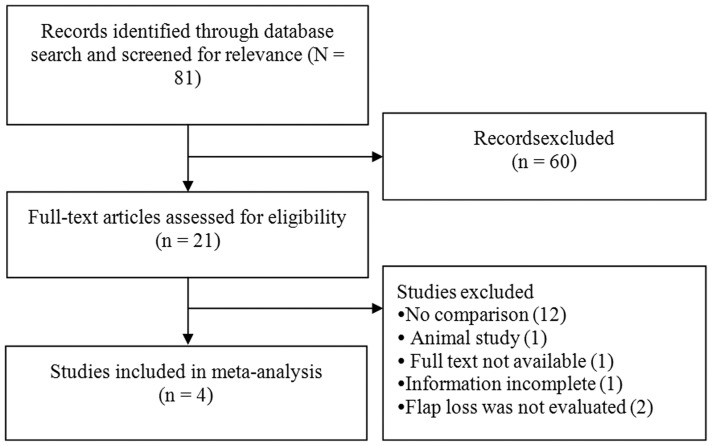
Flow diagram of study selection.

A summary of the literature search results are shown in Figure 1. Briefly, of 81 studies that were identified in the initial search and screened for relevance, 4 meet the criteria for analysis and were included in this study. The characteristics of the 4 retrospective studies included in this meta-analysis are summarized in Table 1.

**Table 1:** Summary of retrospective studies included in the meta-analysis.

1st Author	Year of Publication	Recipient Site	Medication/method of administration	Number of Patients	Age, y§	Males*	Flap Loss-Total*	Flap Loss-Partial*	Hematoma*	Overall Complication Rate
Lighthall [15]	2013	Head and neck	Aspirin only	142	n/a	n/a	7 (4.9)	n/a	10 (7.0)	38%
			Prophylactic heparin/LMWH	25	n/a	n/a	2 (8)	n/a	6 (24)	48%
			Aspirin + prophylactic Heparin/LMWH	23	n/a	n/a	1 (4.3)	n/a	0 (0)	65%
			Heparin drip	16	n/a	n/a	5 (31.3)	n/a	5 (31.3)	100%
			No anticoagulation	184	n/a	n/a	10 (5.4)	n/a	7 (3.8)	24%
Blackburn [16]	2012	Head and neck	Low dose dalteparin, 2500 units	30	63±14	22 (75)	1 (3.3)	1 (3.3)	n/a	n/a
			High dose dalteparin, 5000 units	29	60±14	24 (80)	4 (13.8)	1 (3.4)	n/a	n/a
Ashjian [17]	2007	Head and neck; trunk and breast; upper extremity; lower extremity	325 mg of aspirin daily	245	57 (10–102)	101 (41)	1 (0.4)	6 (2.3)	6 (2.3)	n/a
			5000 units LMWH daily until ambulating	225	54 (3–89)	102 (45)	2 (0.8)	5 (2)	7 (2.9)	n/a
Kroll [18]	1995	Postmastectomy breast reconstruction; head and neck reconstruction	Low-dose heparin bolus (2000–3000 units) and postoperative infusion at a rate of 100–400 units/h for 5–7 days	192	n/a	n/a	2 (1)	n/a	13 (6.8)	n/a
			High-dose heparin infusion at rate of 500–1200 units/h	30	n/a	n/a	3 (10)	n/a	6 (20)	n/a
			Intraoperative bolus of 5000 units heparin	46	n/a	n/a	0 (0)	n/a	3 (6.5)	n/a
			Dextran 40 infusion at rate of 25 ml/h	22	n/a	n/a	6 (27.2)	n/a	2 (9.1)	n/a
			No anticoagulation	227	n/a	n/a	10 (4.4)	n/a	12 (5.3)	n/a

§ Data are presented as mean±standard deviation, or median (range).

*Data are presented as number (percentage).

LMWH, low molecular weight heparin; n/a, not available.

doi:10.1371/journal.pone.0095111.t001

### Outcome Measures

**Figure 2:**
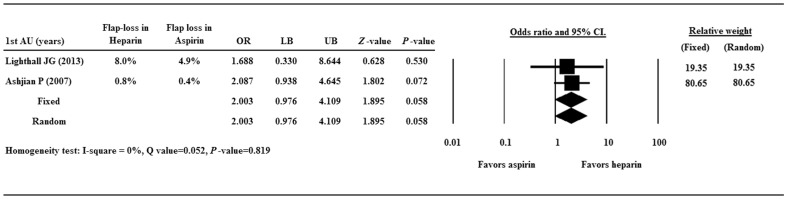
Forest plot showing odds ratios (ORs) of flap lossbetween heparin and aspirin. Data are presented as OR with the 95% confidence interval (CI). A value of *p*<0.05 indicates a statistically significant difference.

Two studies [15],[17] compared aspirin and heparin for the prevention of flap loss, and the ORs of the 2 studies were 1.688 and 2.087, respectively (Figure 2). There was heterogeneity in the combined OR of the 2 studies (Q = 0.052, I^2^ = 0%, *p* = 0.819); therefore a fixed-effects model of analysis was used. The combined OR of 2.003 (95% CI 0.976–4.109, *p* = 0.058) did not indicate any significant difference between heparin and aspirin therapies. The result indicates that the flap loss rate is similar when heparin or aspirin is used as a preventive measure.

**Figure 3:**
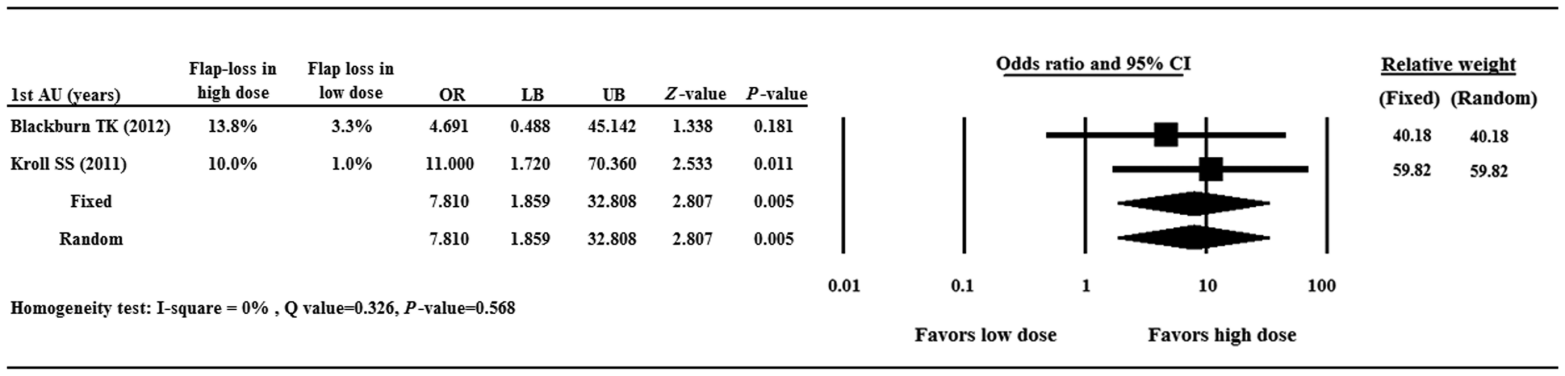
Forest plot showing odds ratio (OR) offlap lossbetween high and low doses of dalteparin or heparin. Data are presented as OR with the 95% confidence interval (CI). A value of *p*<0.05 indicates a statistically significant difference.

Two studies[16],[18] compared the flap loss rate between high and low doses of dalteparin/heparin therapies, and the ORs of the 2 studies were 4.691 and 11.00, respectively (Figure 3).There was no heterogeneity in the combined OR of the 2 studies (Q = 0.326, I^2^ = 0%, *p* = 0.568); therefore a fixed-effects model of analysis was used. The combined OR of 7.810 (95% CI 1.859–32.808, *p* = 0.005) revealed a significant difference between high and low doses of dalteparin or heparin therapies. The result indicates that high dose dalteparin or heparin therapy is associated with a greater flap loss rate than low dose therapy.

## Discussion

The results of this study indicate that heparin and aspirin prophylaxis are associated with similar flap loss rates after free flap transfer. Furthermore, high dose dalteparin or heparin therapy is associated with a greater flap loss rate than low dose therapy.

While thrombotic occlusion is the leading cause of flap failure [4],[5], and surgeon experience is an important factor contributing to flap loss [3], studies have identified other potential factors that may contribute to flap failure. Rubino et al. [19] studied perforator flaps and found that the postoperative flow rate of flap arteries was significantly correlated with flap weight, and that the minimum diameter of veins needed to drain flaps weighting 300, 500, and 900 g was 1.30, 1.50, and 1.75 mm, respectively. In an interesting study of deep inferior epigastric perforator (DIEP) flaps used for breast reconstruction, Santanelli et al. [20] reported that on multivariate analysis nulliparity was the only significant factor associated with partial flap loss and fat necrosis, and that a medial row perforator and a fluid infusion of crystalloid/colloid combined decreased the risk by approximately 11% and 27%, respectively. Ketorolac has been suggested to be effective in preventing microvascular thrombosis in lower extremity reconstruction [21]; however, as Longo and Santanelli [22] have pointed out study methodology can affect results and there is currently no consensus on the role of anticoagulants in preventing microvascular thrombosis.

The ideal anticoagulant for free flap surgery would effectively reduce thrombosis in the pedicle with minimal adverse side effects. In areas of stasis where there is aggregation of fibrin and red cells aggregate venous thrombosis can occur, whereas arterial thrombosis occurs in regions with tortuous flow as a result of aggregation of platelets and thrombin [17]. Most microvascular surgeons use some form of antithrombotic prophylaxis such as heparin, aspirin, dextran, or other antithrombotic agent. However, no clinical reviews so far have conclusively shown any regimen to optimize free flap success. On the other hand, the use of low dose heparin or LMWH is considered mandatory for the prevention of thromboembolism in general surgical procedures. That heparin is effective in preventing thrombosis in general surgical procedures serves as the rationale for its use in microvascular free flap transfer. The intraoperative use of heparin as a bolus and for irrigation is general considered to be safe and advantageous, and the postoperative administration of aspirin is likewise considered safe with similar efficacy to heparin or LMWH. However, these beliefs are generally based on data from animal studies, small retrospective case series, or surgeon's anecdotal experience [1],[4],[5].

Heparin is a glycosaminoglycan which inhibits fibrin formation by increasing the antithrombin mediated inhibition of thrombin and factor Xa. LMWH and low-dose unfractionated heparin are effective in preventing postoperative arterial andvenous thorombosis [9]–[11]. However, the effectiveness of heparin for the prevention of thrombosis with free flap transfer and microvascular anastomosis remains unclear, although it is commonly used for this purpose. In addition to there being few prospective randomized studies, there are various forms of heparin and methods and timings of administration [23],[24].

Some authors have attempted to provide guidance based on their own experience and review of current literature. For example, Conrad et al. [25] developed an algorithm for free-flap thrombosis prophylaxis and failure in which low dose aspirin is given for 2 weeks pre- and postoperatively, and an intraoperative bolus of heparin is administered. Lecoq et al. [13] performed a review of all citations published from 1996 to 2005 regarding thromboprophylaxis for free flap transfer, and concluded that anticoagulation, preferably with heparin, is mandatory for microsurgery.

Two of the studies in this meta-analysis evaluated differences in heparin dosages and administration. Blackburn et al. [16] examined bleeding complications in patients receiving free flap reconstruction for oral and oropharyngeal cancer who received either 2,500 or 5,000 units of dalteparin 12 hours before surgery, and found no difference in bleeding index or bleeding complications between the 2 groups. However, there was a trend (*p* = 0.25 Fisher's exact test) towards a higher rate of flap failure in the high dose group (17%, 4 complete and 1 partial failure) compared with 7% (1 complete and 1 partial failure) in the low dose group. In the other study, Kroll et al. [18] examined the flap loss rate and hematoma formation in 5 groups of patients receiving free flap transfers for head and neck reconstruction: 1) low-dose heparin bolus (2000–3000 units) and postoperative infusion at a rate of 100–400 units/h for 5–7 days; 2) high-dose heparin infusion at rate of 500–1200 units/h; 3) intraoperative bolus of 5000 units heparin; 4) dextran 40 infusion at rate of 25 ml/h; and 5) no anticoagulation. The flap loss rates in the no anticoagulation, low-dose heparin, bolus heparin, high-dose heparin, and dextran 40 groups were, respectively, 4.4%, 1.0%, 0%, 10%, and 27.2%, and the thrombosis rates were, respectively, 6.1%, 2.1%, 2,2%, 13,3%, and 31.8%.

Aspirin is the most widely used platelet inhibitor, and inhibits thromboxane synthesis by antagonizing cyclooxygenase. The use of aspirin for preventing thrombosis in microvascular surgery is based on is ability to inhibit arterial occlusions [5]. Chien et al. [26] reported that subcutaneous heparin 5000 U twice per day and aspirin (325 mg orally per day) were equivalent in preventing flap loss in patients undergoing head and neck reconstruction, and the safety profiles were similar.

There were 2 studies included in this meta-analysis that compared the use of aspirin and heparin for prophylaxis. In a retrospective analysis, Lighthall et al. [15] compared the results of free tissue transfers for head and neck reconstruction in which 184 cases received no postoperative prophylaxis, 142 received aspirin, 48 received LMWH with or without other agents, and 16 received a heparin drip. The overall flap loss rate was 6.4%, while the loss rates of the different regimens were 5.4% (no agent), 4.9% (aspirin), 8.0% (prophylactic heparin/LMWH), 4.4% (combination therapy), and 31.3% (heparin drip). The loss rate in the heparin drip group was statistically greater than in the other groups, but there were no other between-group differences. While there were significantly more complications in the aspirin group compared with no prophylaxis, there was no significant difference in bleeding complications or the flap failure rate between the groups that received aspirin and no prophylaxis. In the other study in which aspirin was examined, Ashjian et al. [17] compared the results of prophylaxis with 325 mg of aspirin administered daily for 5 days postoperatively (n = 260) with that of 5000 units of LMWH daily until ambulating (n = 245) in patients undergoing free flap reconstruction for oncological defects. No statistically significant differences between the groups were found the occurrence of microvascular thrombosis, partial or total flap loss, hematoma, bleeding, deep vein thrombosis (DVT), pulmonary embolism, and death.

The primary outcome measure of this analysis was flap loss; however, the occurrence of bleeding complications and hematoma formation are important considerations when performing prophylaxis for thrombosis after free flap transfer. Khouri et al. [3] examined 493 free flaps performed by 23 surgeons and reported that the rate of postoperative thrombosis requiring re-exploration was significantly lower when subcutaneous heparin was administered in the postoperative period. However, the flap failure rate (4.1%) was not affected by the type of postoperative antithrombotic regimen used. A multicentertrial investigated the use intraluminal irrigation with recombinant human tissue factor pathway inhibitor (rhTFPI; SC-59735) during microvascular anastomosis in free flap reconstructive surgery [27]. rhTFPI at a concentration of 0.05 or 0.15 mg/ml (low-dose or high-dose group, respectively) or heparin at a concentration of 100 U/ml was used to irrigate the vessel after anastomoses and before blood was reestablished.The flap failure rates were 2%, 6%, and 5% for the low-dose rhTFPI, high-dose rhTFPI, and heparin groups (*p* = 0.069), as were the rates of intraoperative revisions of vessel anastomoses (11%, 12%, 13%, respectively) and postoperative thrombosis (8%, 8%, 7%, respectively). The postoperative wound hematoma rate was significantly lower in the low-dose rhTFPI group (3%) than in the high-dose rhTFPI group (8%) and the heparin group (9%) (*p* = 0.040). Eley et al. [12] examined dalteparin prophylaxis in patients undergoing free flap transfer for head and neck reconstruction using 4 different dosing regimens: 2,500 IU once per day, 5,000 IU once per day, 5,000 IU twice per day, and 7,500 IU or more once per day, and reported that the percentage of bleeding complications in the 4 groups were 5%, 63%, 21%, and 11%, respectively, and the number of unexplained hematomas in the 4 groups were 0, 4, 2, and 0, respectively. Dhiwakar et al. [28] reported a significant increase in hemorrhage and hematoma after excision of head and neck lesions with either primary repair or local flap closure when patients received perioperative aspirin.

There are limitations to this meta-analysis, including that fact that all studies were of a retrospective nature. The number of studies included was small, and only 2 studies were used for each part of the analysis. The statistical significance level for the analysis in this study was set as *p*<0.05, and the statistical assessment is weak due to the small sample size.

## Conclusions

In conclusions, heparin and aspirin prophylaxis are associated with similar flap loss rates after free flap transfer, and importantly, high dose dalteparin or heparin therapy is associated with a greater flap loss rate than low dose therapy. These conclusions should be interpreted with caution considering the limitations of the including a small number of retrospective studies and a somewhat weak statistical significance. More studies are required to confirm or repute the findings of this analysis.

## Supporting Information

Checklist S1 (DOC)
